# Localized instance fusion of MRI data of Alzheimer’s disease for classification based on instance transfer ensemble learning

**DOI:** 10.1186/s12938-018-0489-1

**Published:** 2018-05-02

**Authors:** Xiaoheng Tan, Yuchuan Liu, Yongming Li, Pin Wang, Xiaoping Zeng, Fang Yan, Xinke Li

**Affiliations:** 1 College of Communication Engineering, Chongqing University, Shapingba District, Chongqing, 400044 China; 2 Department of Medical Image, College of Biomedical Engineering, Third Military Medical University, Chongqing, 400038 China

**Keywords:** Alzheimer’s disease, Magnetic resonance imaging, Instance transfer learning, Classification, Localized instance fusion

## Abstract

**Background:**

Diagnosis of Alzheimer’s disease (AD) is very important, and MRI is an effective imaging mode of Alzheimer’s disease. There are many existing studies on the diagnosis of Alzheimer’s disease based on MRI data. However, there are no studies on the transfer learning between different datasets (including different subjects), thereby improving the sample size of target dataset indirectly.

**Methods:**

Therefore, a new framework method is proposed in this paper to solve this problem. First, gravity transfer is used to transfer the source domain data closer to the target data set. Secondly, the best deviation between the transferred source domain samples and the target domain samples is searched by instance transfer learning algorithm (ITL) based on wrapper mode, thereby obtaining optimal transferred domain samples. Finally, the optimal transferred domain samples and the target domain training samples are combined for classification. If the source data and the target data have different features, a feature growing algorithm is proposed to solve this problem.

**Results:**

The experimental results show that the proposed method is effective regardless of different kernel functions, different number of samples and different parameters. Besides, the transferred source domain samples by ITL algorithm can enlarge the target domain training samples and assist to improve the classification accuracy significantly.

**Conclusions:**

Therefore, the study can enlarge the samples of AD by instance transfer learning, thereby being helpful for the small sample problems of AD. Since the proposed algorithm is a framework algorithm, the study is heuristics to the relevant researchers.

## Background

Alzheimer’s disease is seriously hazardous and the diagnosis is important. MRI is an important neuroimaging, so based on MRI data to achieve AD diagnosis is an effective way [1–3]. At present, there have been many studies about diagnosis of AD based on MRI data [4–18]. Machine learning algorithms overcomes limitations of traditional methods by mining the information among the MRI data for diagnosis of disease. Therefore, it has been widely used in the diagnosis of AD based on MRI [6–14]. Some researchers have studied the MRI of AD patients with traditional machine learning methods and deep learning methods [6–9]. Some studies are based on the MRI structural imaging [4, 11–13, 17] to find the difference between AD and normal people, and others are based on the MRI functional imaging [12] and brain network [15] to distinguish between AD and normal people.

These studies have shown that machine-learning method is effective for AD classification, but most of them are based on the public data set, not for the specific people. In fact, for different regions [19–22], ethnicities [22–24], etc., the characteristics of AD is different, so it is necessary and meaningful to develop classification method for the specific people (target subjects, or target samples).

However, because AD is concealed, slow, non-lethal, sample collection is very difficult, and the number of samples is often less [3, 25–28]. According to the principle of machine learning, the small number of samples is likely to lead to inadequate training and over-fitting. Therefore, there is conflict between small samples and good classification performance (adequate training).

In fact, there are some public datasets available (e.g., The Alzheimer’s Disease Neuroimaging Initiative, ADNI). Although the subjects within the datasets are different from those in the target dataset, all of them are about AD, so they are correlative. The information within the dataset are helpful for pre-training of the classifier to replace the random initialization of the classifier.

Therefore, how to use these public data sets to effectively improve the accuracy of classification of target subjects is a key problem. Recent studies about machine learning show that transfer learning can help to solve this problem. Transfer learning has the advantage of transferring the well-learnt knowledge from the related work to facilitate an improved learning result of one task [29]. It has been applied to solve the problem of small number of samples [29–33]. However, there is few studies on transfer learning for classification of AD. Cheng et al. [3] used the data of AD and normal controls (NC) samples as source domain data to test MCI-C and MCI-NC samples and achieved good results. After that (2017), they also proposed a multi-domain transfer learning framework for early diagnosis of AD [34]. Filipovych et al. [35] have explored the potential of semi-supervised pattern classification to provide image-based biomarkers in the absence of precise diagnostic information of some individuals. They employed semi-supervised support vector machines (SVM) for classifying MR brain images of patients with uncertain diagnoses. Young et al. [37] introduced Gaussian process (GP) classification to the problem. GP can integrate multimodal data, The GP approach aided combination of different data sources by learning parameters automatically from training data via type-II maximum likelihood, which they compared to conventional method based on cross validation and an SVM classifier. The GP has a substantially higher accuracy than that using any individual modality or using a multi kernel SVM. Filipovych et al. and Zhang et al. [35, 36] considered the heterogeneity of MCI to construct semi-supervised classification or regression models (where MCI subjects are regarded as unlabeled samples), which shows that using information of MCI can help improve the performance of classifying or estimating AD patients from NCs. Guerrero et al. [38] proposed a framework to learn a joint low dimensional representation of brain MR images, acquired either at 1.5 or 3 T. In this manifold subspace, knowledge can be shared and transferred between the two distinct but related datasets. Huang et al. [39] proposed a transfer learning approach for diagnosis of brain connectivity networks of Alzheimer’s disease from functional magnetic resonance image data.

The relevant studies above show the effectiveness of the transfer learning for classification of AD. However, these papers do not study how to use other related data sets to improve the classification accuracy of the target data set based on transfer learning [3, 34–39]. In fact, the number of samples is a key bottleneck problem, no matter it is a single mode or multimodal, it is traditional machine learning or deep learning, it is shape features or texture features or brain network characteristics. Since there are some similar public data sets, it is necessary to study the effective transfer learning to make full use of these related data sets to improve the classification accuracy of the target data set. Besides, most of the existing relevant studies focus on the transferring of the parameters of classifiers, and cannot transfer the samples, thereby obtaining more training samples. Therefore, it is also necessary to study the effective transfer learning to make full use of these related data sets to enlarge target training samples.

Based on the analysis above, a method based on transfer learning is proposed to meet the requirements. First, gravity transfer is used to make a rough migration of the source domain samples to the target domain samples. Secondly, the best deviation between the source domain samples and the target domain samples is searched based on wrapper mode and the optimal deviation is obtained between the source and the target domain. Finally, the transferred source domain samples plus optimal deviation are used for classification by combining with the target training samples.

## Methods

### Subjects/database

In order to verify the validity of the algorithm, two related but different data sets are used to verify the algorithm. One of the data sets is a publicly accessible ADNI database (http://adni.loni.usc.edu/), and the other data set is the one from us (called local data). The local data is the target of classification of AD, so it is called target data (target samples; target subjects); the ADNI database include more samples of AD, but the subjects are different from those of the local data.

#### The publicly accessible ADNI database (ADNI data)

The ADNI database is organized into three Microsoft excel files in ADNI, which are IDA_MR_Metadata_Listing, idaSearch_5_04_2015 and UCSFSNTVOL. The samples in the publicly accessible ADNI database had only two image features and had not been processed with feature selection. The two features of the data set were the volumes of the left and right parts of the hippocampus. The total number of samples in the data set was 951, consisting of two classes of samples: NC and AD. The number of NC samples was 540 and the number of AD samples was 411. The age distribution ranges of the two classes of samples were all 65–85 years old. The MRI sequence used is T2 dual echo sequence at 1.5 or 3.0 T; the image size is 256 × 256 × 170 voxels with the voxel size of approximately 1 mm × 1 mm × 1.2 mm. The image scanner is a GE Medical Systems scanner. With the SPM8 package and the VBM8 toolbox, two features are extracted from the MR images, which are the volumes of left and right hippocampus. The feature data is stored as excel file in the ADNI.

To simplify the analysis, the samples were roughly divided into two classes: NC and AD. Moreover, the numbers of the two classes of samples were the same to eliminate the effects of unbalanced samples. The number of AD samples was 411 or less, so the number of samples of different classes was 411. The two classes of samples were within similar age distribution ranges of 65–85 years old. Relevant, brief information about the ADNI dataset is shown in Table 1.

**Table 1:** Basic information about the ADNI dataset

Class	Number	Age range (years)	Mean age (years)	Age standard deviation	Men/women
NC	411	65–85	76.092	4.696	185/226
AD	411	65–85	75.503	7.245	198/213

#### The AD dataset from us (local data)

The local AD dataset were chosen with preprocessing and feature extraction. The samples had 32 image features including two shape features (the volumes of the left and right parts of the hippocampus) and 30 texture features. The dataset consists of structural T1 MR images of 90 subjects. Because the number of the effective AD samples is small, the number of samples for each class is 30 for balance. These images were acquired by GE 1.5T Signa scanner at the Southwest Hospital, China. The spoiled gradient-recalled (SPGR) volumetric T1-weighted pulse sequence was used with the following parameters, optimized for maximal contrast among gray matter, white matter, and CSF: TE = 5 ms, TR = 25 ms, flip angle = 40, NEX = 1, slice thickness = 1.5 mm/0 mm interslice. The individuals for the study were selected by neuroradiologists. No participant has a neurological disease and all have similar educational level.

The experimental data have been uploaded to the public cloud disk of our laboratory (https://pan.baidu.com/s/1dmsUfk). It is convenient for readers and editors to view them.

### Methods

In order to combine two datasets, the proposed method was named as Instance Transfer Learning (ITL) which can effectively transfer the source data to the target data. First, gravity transfer is used to make a rough migration of the source domain samples to the target domain samples. Then the best deviation between the source domain samples and the target domain samples is searched based on wrapper mode and the best deviation between the source and the target domain is obtained. The wrapper mode here means the evaluation criterion for searching the optimal deviation, which is the classification accuracy of validation set. If the features of the source data and the target data are same, ITL algorithm can be used to conduct instance transfer learning. However, if the features of the source data and the target data are not same, ITL algorithm is not enough. The common features between the source data and the target data are chosen. Based on the features, ITL algorithm is used to conduct instance transfer learning. Suppose the number of features of target data is *N*_*t*arg*et*_, and the number of the common features is *N*_*com*_, feature growing algorithm is designed to obtain the (*N*_*t*arg*et*_ − *N*_*com*_) features, thereby transferring the source data to those close to the target data. The source data after transformation have the same features with the target data. After that, ensemble learning is conducted for improving classification accuracy.

In the paper, the ADNI data is the source domain (SD) data, the local data is the target domain (TD) data. The target data set is divided into training sets and test sets, denoted as TD_train and TD_test. One part of training set is for training the classifier; another part of training set is for validating and searching deviation.

The number and features information of the data set sample is shown in Table 2. More detailed feature information, please see Appendix.

**Table 2:** Basic information about the ADNI dataset

Database	Class	Number of samples	Number of features	Features information
ADNI (source domain data)	AD	411	2	2 shape features
	NC	411	2	
Local (target domain data)	AD	30	32	2 shape features and 30 texture features
	NC	30	32	

#### Instance transfer learning (ITL)

Due to the small number of deviation candidates, an exhaustive approach is used to find the optimum deviation for the candidates. The fitness function of the deviation is the maximization of classification accuracy. The fitness function can be described as follows.

1 $$Dc_{i} = \arg \left[ {\text {max}\left( {acc(\hat{y},y_{label} )} \right)} \right]$$

where $\hat{y}$ is the output predicted by the model, and *y*_*label*_ is the label of samples.

**Fig. 1:**
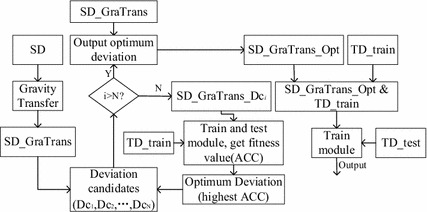
The flow chart of the proposed algorithm (ITL)

Assuming the number of the candidate deviations is N. The main procedures are shown in the Fig. 1.

In Fig. 1, the TD_train is divided into two parts, one part is used to guide the SD_GraTrans data migration to the target domain and train model with the migrated source domain data (SD_GraTrans_Dc_i_). The other part is used to validate the model and obtain the fitness value of the deviation candidate. The cross-validation algorithm used here is to leave one-out cross-validation method. In the figure, SD_GraTrans_Opt is obtained by SD_GraTrans plus the output optimum deviation. SD_GraTrans_Opt&TD_train is obtained by combining SD_GraTrans_Opt and TD_train.

The pseudo code of ITL algorithm is shown as follows. 

#### Feature growing algorithm (FGA)

In the instance transfer learning process based on ITL, only some features (left and right hippocampus) are same between the source data and target data (local data), and the texture features are not used. The texture features are needed to be added to the source dataset. According to similarity principle, the Euclidean distance are used as a similarity criterion between the source samples and the target samples. The fitness function of the Euclidean distance is as follows.

2 $${\text{Distance}} = \sqrt {\sum\limits_{i = 1}^{n} {(Xs_{i} - Xt_{i} )^{2} } }$$

where Distance is the Euclidean distance between the *Xs* and *Xt*, n is the number of features.

In this paper, the Euclidean distance criterion is used to match the texture features from the target domain to the source domain samples. The fitness function F is defined as:

3 $$F_{i} = \arg \left\{ {\hbox{min} \left[ {{\text{Distance}}\left( {Xs_{i} ,Xt_{j} } \right)} \right]} \right\},\quad{\text{where}}\;j \in \left[ {1,N} \right]$$

where *F*_*i*_ means the fitness value of the *i*th sample of source data (ADNI data), Distance(*Xs*_*i*_, *Xt*_*j*_) means the distance between *Xs*_*i*_ and *Xt*_*j*_, N is the number of the target data (local data train), *Xs*_*i*_ means the *i*th sample of source domain, *Xt*_*j*_ means the *j*th sample of target domain.

Here, the sample selection algorithm is used, where the confidence criterion is used to select the source samples. TD_train means the training set in the target data. According to the Euclidean distance criterion, the confidence criterion calculates the Euclidean distance between each sample in the source domain and all the TD_train samples. The N samples closest to the source domain are found from the TD_train, and the closest sample’s label is used as the label of the corresponding sample in the source domain data. Then, the label of the sample is compared with the labels of other N − 1 samples chosen from TD_train, and the number of the samples with the same label is counted. The higher the number is, the higher the confidence is.

**Fig. 2:**
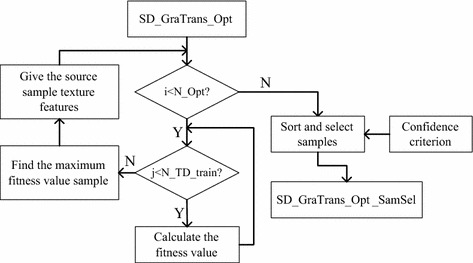
The flow chart of feature growing algorithm

In the part, assuming that the number of SD_GraTrans_Opt is N_Opt, the number of target data train sets is N_TD_train. The main procedure is shown in Fig. 2.

#### Ensemble learning algorithm (ELA)

**Fig. 3:**
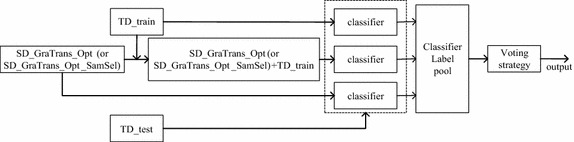
The flow chart of ensemble learning algorithm

Based on the principle of the ITL, ensemble learning is used to enhance the stability of the classification model. The flow chart is shown in Fig. 3.

When the FGA algorithm is not used, SD_GraTrans_Opt is used as input for Ensemble learning. When the FGA algorithm is used, SD_GraTrans_Opt_SamSel is used as input for Ensemble learning. As can be seen from the figure, an optimal deviation can generate three classifiers, and the classifiers obtained from the deviations are combined to get the ensemble classifier. Finally, the ensemble classifier is tested with the TD_test and the final prediction results are obtained.

## Results

### Experimental conditions

In the paper, the experimental operating system platform was the Windows, version 7, 64-bit operating system, and the memory size was 128G. The data processing was completed in MATLAB, version 2014a. In this paper, leave-one-out are used as cross-validation method. In the classification process, the classifier is support vector machine (SVM). The kernel functions of SVM is linear kernel and RBF kernel.

Several groups of experiments are organized to verify the performance of the proposed method in this paper. In the 1st group of experiments, under the condition where the features are same between source data and target data, the performance of the ITL + ELA algorithm is shown and compared. In the 2nd group of experiments, under the condition where the features are not same between source data and target data, the performance of the ITL + FGA + ELA algorithm is shown and compared.

### Evaluation of ITL + ELA algorithms in the case of same features

In this section, support vector machine (SVM) is used as classifier. Different parameters of SVM are involved including different kernel functions and different kernel function parameters. The experimental results are recorded in Table 3. Here, SD_GraTrans_Opt means the method by ITL algorithm; SD_GraTrans_Opt+TD_train means the SD_GraTrans_Opt with TD_train; SD_GraTrans+TD_train means the SD_GraTrans with TD_train; TD_train means the train set (just two shape features) in the target data; SD+TD_train means the SD with TD_train.

**Table 3:** Evaluation of ITL + ELA algorithms in the case of same features

	Parameter		SD_GraTrans_Opt +TD_train (%)	SD_GraTrans +TD_train (%)	TD_train (%)	SD+TD_train (%)
	Cost	Gamma				
SVM (linear)	2	0.03125	*83.33*	78.33	76.67	50
	1.5	0.03125	*83.33*	76.67	76.67	50
	1	0.03125	*83.33*	76.67	76.67	50
	1	0.3125	*80*	76.67	76.67	50
	1	0.003125	*83.33*	76.67	76.67	50
SVM (RBF)	2	0.03125	*83.33*	76.67	76.67	50
	1.5	0.03125	*83.33*	76.67	76.67	50
	1	0.03125	*83.33*	78.33	76.67	50
	1	0.3125	*83.33*	78.33	76.67	50
	1	0.003125	*81.67*	66.67	60	50

The italicized data represents the highest classification accuracy under the same experimental conditions

It can be seen from Table 3, the classification accuracies with SD_GraTrans_Opt+TD_train are always better than those with TD_train regardless of different parameters and kernel types. It means that the proposed ITL algorithm is effective. The classification accuracies with SD_GraTrans_Opt+TD_train are always better than those with SD+TD_train regardless of different parameters and kernel types. The results demonstrate that simply combination of the source data and target data cannot work well. The classification accuracies with SD_GraTrans_Opt+TD_train are always better than those with SD_GraTrans+TD_train regardless of different parameters and kernel types. The results mean that simple transfer (gravity transfer) is not enough. Compared with different parameters, it was found that the parameters have no apparent effect on the accuracy. Compared with different kernel types, it was found that the kernel types have no apparent effect on the accuracy.

In this section, the effect of different samples of the TD is studied. The method of leave-one-out (LOO) is used for cross validation, and the final classification accuracy is calculated. Each experiment is repeated ten times, and the results obtained as follows.

From the table, it can be seen that regardless of the sample size the proposed algorithm (SD_GraTrans_Opt+TD_train) achieves the highest classification accuracy under different kernel functions. For example, in the case of linear kernel functions when the number of samples is 60, the classification accuracy of the target domain is 83.33%. Its classification accuracy is higher than TD_train (76.67%) and SD_GraTrans+TD_train (76.67%). Besides, with the number decreases, the classification accuracies of TD gradually decreases. The result means that more training samples will be helpful for classification. However, it is very hard to collect large number of samples, especially for AD. As for the proposed method (SD_GraTrans_Opt+TD_train), the classification accuracy is not affected by the number of the samples of TD. Therefore, it is feasible to make use of relevant large-scale dataset for improving the accuracy of local dataset.

**Fig. 4:**
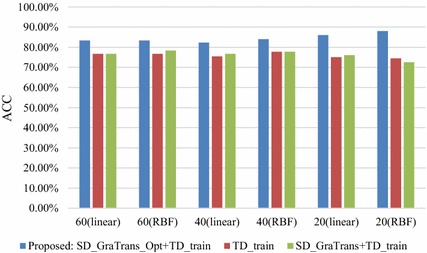
The classification accuracy of the target dataset under different conditions

Figure 4 shows the results of Table 4 visually.

**Table 4:** The results of the target domain in the case of different sampling

Number of samples in TD	Kernel type	SD_GraTrans_Opt+TD_train (Mean, std)	SD_GraTrans+TD_train (Mean, std)	TD_train (Mean, std)
60	Linear	*(83.33*%, *0)*	(76.67%, 0)	(76.67%, 0)
	RBF	*(83.33*%, *0)*	(78.33%, 0)	(76.67%, 0)
40	Linear	*(82.29*%, *0.0419)*	(76.67%, 0.0504)	(75.42%, 0.0601)
	RBF	*(84*%, *0.0129)*	(77.75%, 0.0184)	(77.75%, 0.0249)
20	Linear	*(86*%, *0.0615)*	(76%,0.0658)	(75%, 0.0882)
	RBF	*(88*%, *0.0422)*	(72.5%, 0.0795)	(74.5%, 0.1066)

The italicized data represents the highest classification accuracy under the same experimental conditions

It can be seen that from the figure that the proposed algorithm has the best classification accuracy compared with other algorithms no matter the number of the samples in the TD.

Table 5 summarizes the false detection rate and false positive information in the case of Table 4. In the Table 5, FP means false positive, FDR means false detection rate.

**Table 5:** The false positive and false detection rate information

Number of samples in TD	Kernel type	SD_GraTrans_Opt+TD_train		SD_GraTrans+TD_train		TD_train	
		FP (%)	FDR (%)	FP (%)	FDR (%)	FP (%)	FDR (%)
60	Linear	6.67	9.52	10	15	20	21.43
	RBF	6.67	9.09	10	15	20	21.43
40	Linear	5	7.14	10	16.67	15	18.75
	RBF	20	22.22	20	25	20	21.05
20	Linear	10	11.11	10	11.11	30	25
	RBF	0	0	10	11.11	30	27.27

As can be seen from the table, under the same conditions, the proposed method has a lower false positive than the other methods. In most cases, the false detection rate is also lower than other methods. Therefore, the effectiveness of the proposed algorithm is validated. In addition, compared with the result of simple migration (SD_GraTrans+TD_train), SD_GraTrans cannot be directly used to supplement TD data.

### Evaluation of ITL + FGA + ELA algorithms in the case of different features

As described above, when the features between source data (SD) and target data (TD) are not same, the ITL is not enough. The solution is as follows: first, the SD is transformed to TD by ITL based on the common features. Secondly, transformed SD is transformed to TD by FGA, to enlarge the features that are the same as those of TD. In this section, different conditions are considered, including different number of samples of TD, different kernel types, and different sub-classifiers.

**Fig. 5:**
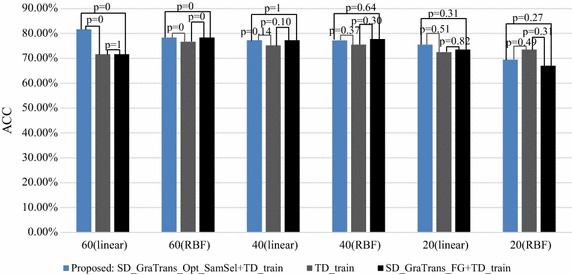
Classification accuracy of different algorithms when the number of samples in TD changes

The number of sample selections for source data is 411 (half of the number of source data samples). As described above, the optimal deviations are not unique. So, different numbers of optimal deviations are considered here, and the best number is 25. Each experiment is repeated ten times, the results can be found in the Tables 6, 7, and Fig. 5. In Table 6, the SD_GraTrans_Opt_SamSel means the SD_GraTrans_Opt after adding texture feature by FGA algorithm and sample selection. The SD_GraTrans_FG means the SD_GraTrans after adding texture feature by FGA algorithm. SD_GraTrans_Opt_SamSel+TD_train means the SD_GraTrans_Opt_SamSel with the TD_train. SD_GraTrans_FG+TD_train means the SD_GraTrans_FG with the TD_train. Here, the TD_train has all the features.

**Table 6:** The results of the target domain in the case of different sampling after feature growing

Number of samples in TD	Kernel type	SD_GraTrans_Opt_SamSel+TD_train (Mean, std)	SD_GraTrans_FG+TD_train (Mean, std)	TD_train (Mean, std)
60	Linear	*(81.67*%, *0)*	(71.67%, 0)	(71.67%, 0)
	RBF	*(78.33*%, *0)*	(78.33%, 0)	(76.67%, 0)
40	Linear	*(77.29*%, *0.0505)*	(77.29%, 0.0376)	(75.21%, 0.0538)
	RBF	(77.25%, 0.0362)	*(77.75*%, *0.0416)*	(75.5%, 0.0705)
20	Linear	*(75.5*%, *0.1322)*	(73.5%, 0.1292)	(72.5%, 0.1112)
	RBF	(69.5%, 0.1707)	(67%, 0.1844)	*(73.5*%, *0.0747)*

The italicized data represents the highest classification accuracy under the same experimental conditions

**Table 7:** The false positive and false detection rate information

Number of samples in TD	Kernel type	SD_GraTrans_Opt_SamSel+TD_train		SD_GraTrans_FG+TD_train		TD_train	
		FP (%)	FDR (%)	FP (%)	FDR (%)	FP (%)	FDR (%)
60	Linear	10	13.04	16.67	26.32	20	22.22
	RBF	6.67	11.76	13.33	20	23.33	24.14
40	Linear	15	20	45	47.37	20	23.53
	RBF	15	15.79	20	20	15	15
20	Linear	10	11.11	10	11.11	10	11.11
	RBF	30	27.27	40	33.3	20	22.22

Seen from Table 6, when the added texture features are used for classification, the accuracy of the classification in all cases decreased (compared with the Table 4). A possible reason is that the added texture features contain a lot of redundant information, which leads to the decrease of classification accuracy. However, in the case of linear kernel function, the transferred samples combining with TD training samples is still better than only TD training samples in terms of classification accuracy. In the case of RBF kernel function, with the number of the target dataset decrease, the classification accuracy of proposed algorithm and SD_GraTrans_FG+TD_train are obviously decreased. The classification accuracy of TD_train has decreased too. But the classification accuracy by the proposed algorithm is still better than that by TD_train. The results mean that the proposed algorithm can effectively transfer the other source dataset to improve the classification accuracy of the target dataset.

Figure 5 shows the classification accuracy of different algorithms when the number of samples in TD dataset changes.

From Fig. 5, we can see that, in most cases, the classification accuracy of the proposed algorithm is higher than SD_GraTrans_FG+TD_train and TD_train in significance level. After the source samples are simply migrated, the model trained by SD_GraTrans_FG with TD_train (SD_GraTrans_FG+TD_train) is not worse than the TD_train. It can be concluded that the proposed algorithm is very effective for the transferring of source samples. Significant differences between the different algorithms are also shown in the figure.

Table 7 summarizes the false detection rate and false positive information in the case of Table 6. In Table 7, FP means false positive, FDR means false detection rate.

As seen from the table, under the same conditions, the proposed method has a lower false positive than the other methods. In most cases, the false detection rate is also lower than other methods. Therefore, the data set after feature growth still has a good effect, which indirectly verifies the effectiveness of the proposed algorithm.

As described above, the deviation is possibly not unique. Therefore, the number of the sub-classifiers are explored here. The number of samples are 60. Every deviation generates a sub-classifier, and all the sub-classifiers form an ensemble classifier. Each experiment is repeated ten times, the results obtained as follows.

As can be seen from Table 8, in the case of linear kernel functions, when the ensemble classifier is composed of 25 deviations, the classification accuracy of the proposed algorithm (81.67%) is higher than TD_train (71.67%) and SD_GraTrans_FG+TD_train (71.67%) respectively. As the number of sub-classifiers decrease, this rule is always maintained and the classification accuracy of the proposed algorithm has not changed significantly. However, in the case of RBF kernel function, with the decrease of the number of deviations, the classification accuracy of proposed algorithm decreases. But the classification accuracy is still better than that only with TD_train obviously.

**Table 8:** The influence of the number of classifiers on classification results

Number of sub-classifiers (Deviations)	Kernel	SD_GraTrans_Opt_SamSel+TD_train (Mean, std)	SD_GraTrans_FG + TD_train (Mean, std)	TD_train (Mean, std)
25	Linear	*(81.67*%, *0)*	(71.67%, 0)	(71.67%, 0)
	RBF	*(78.33*%, *0)*	(78.33%, 0)	(76.67%, 0)
10	Linear	*(81.67*%, *0.0079)*	(79.67%, 0.0189)	(71.67%, 0)
	RBF	(74.33%, 0.0161)	*(77.5*%, *0.0425)*	(76.67%, 0)
5	Linear	*(81.33*%, *0.0070)*	(80%, 0.0192)	(71.67%, 0)
	RBF	(74.67%, 0.0205)	(76.33%, 0.0362)	*(76.67*%, *0)*

The italicized data represents the highest classification accuracy under the same experimental conditions

As described above, the proposed FGA algorithm can expand the features based on transferred samples and target samples. The figures and tables above show its effectiveness.

**Fig. 6:**
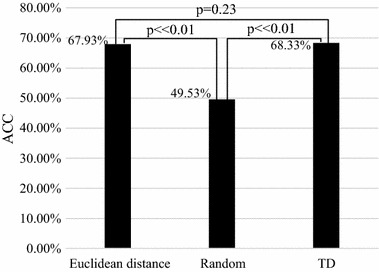
Comparison of texture feature classification in different cases

Figure 6 shows the grown features by the FGA algorithm are close to those of the target samples (p = 0.23) and quite different from those by random growing (p ≪ 0.01). The features of the target samples are different from those by random growing (p ≪ 0.01) too. The result means that the ITL and FGA algorithms are effective. Here, the number of TD samples is 60; kernel type is linear; it can be seen that the classification accuracy of texture features by FGA (67.93%) is higher than that by random growing (49.53%) significantly. Its classification accuracy is very close to the classification accuracy of TD’s texture feature (68.33%). According to the p-values, there is no significant difference between the texture features growing by the FGA algorithm and the texture features of TD. Both of them have significant differences with the texture features by random growing. The result shows that the FGA algorithm is effective.

## Discussion

AD is a serious social problem. For different areas, ethnicity, etc., the characteristics of AD are different. Therefore, the diagnosis of Alzheimer’s disease aiming at local people is very necessary. On the other hand, due to concealed, slow, non-lethal and other characteristics of AD, the sample collection is very difficult, so the number of samples is often small and dispersive. According to the principle of machine learning, small number of samples is likely to lead to inadequate training and over-fitting. Therefore, there is a big problem about how to obtain high efficiency based on small samples. In fact, there exists some public databases containing large relevant samples. Therefore, it is very necessary to study how to make use of the public data to improve the classification accuracy of target data.

In this paper, instance transfer learning (ITL) algorithm was proposed to solve this problem. The samples in SD data can be transformed to target data based on the deviations and generated transferred SD data by ITL algorithm. The transferred SD data can improve the classification accuracy by combining the training set of the target data. Here, ensemble-learning algorithm (ELA) is involved. When the features between the SD data and target data are same, the ITL + ELA can deal with the process. When the features between the SD data and target data are different, the common features between the SD data and target data are dealt with the ITL algorithm; the other features are generated by the FGA algorithm; classification is conducted by ELA algorithm.

The experimental results are positive. Regardless of the number of TD samples and the number of sub-classifier and kernel types, the ITL algorithm in this paper have achieved better results than those by using local data alone. In some cases [such as Table 3, the classification accuracy can be improved up to 13.5% (from 74.5 to 88%)]. In case of linear kernel function of SVM, our proposed method has a better performance than that of TD_train and SD_GraTrans+TD_train in all cases. However, after the feature growing, the classification accuracy did not achieve better results (compared Table 6 with Table 4). It does not mean adding of FGA algorithm is invalid. First, judging from the target dataset itself, the accuracy of classification decreases from two features to multiple features. The possible reason is that texture features have some interference with the classification of volume features. For example, after adding texture features, the classification accuracy of the target dataset decreased by 5% (from 76.67 to 71.67%). However, the proposed algorithm only decreases by 1.66% (from 83.33 to 81.67%), which directly reflects the validity of the FGA algorithm and indirectly reflects the validity of the ITL algorithm. In addition, it is unfair to directly compare the accuracy between Tables 4 and 6. The accuracy change is caused by many factors, such as the different features.

Although there are literatures related to the use of transfer learning for AD diagnosis, there is no research about how to use other datasets (subjects, or samples) to improve the classification accuracy of the target dataset. Therefore, they are completely irrelevant to this paper. In fact, the sample size is a key bottleneck problem, regardless of single mode or multimodal, traditional machine learning or deep learning, shape features or texture features or brain network characteristics. The current relevant literatures always are based on some specific target dataset (public or collected by self), but usually the sample size is small, especially for the datasets collected by those authors. However, according to theory of statistical learning, small sample size always leads to insufficient training of classifier and overfitting. Therefore, it is necessary to study the effective transfer learning to make full use of these related data sets to improve the classification accuracy of the target data set. This is also the main motivation and value of this paper. Besides, current relevant transfer learning algorithms focus on transfer the parameters of the pre-trained model rather than the source domain samples themselves, so they cannot obtain transferred samples and expand the target samples. As we known, for the small sample problems, people yearn for obtaining more samples for subsequent statistical analysis, and so on. The method in this manuscript can solve this problem to some extent.

## Highlights

The main contributions and innovations of this paper can be stated as follows:

1. This paper proposed an instance transfer learning algorithm for classification of Alzheimer’s disease.
2. The instance transfer learning algorithm can deal with the situation when the features between source data and target data are different.
3. The instance transfer learning algorithm can transfer the source samples to target samples, and obtain the transferred source samples, thereby enlarging the target samples.
4. Representative public dataset—ADNI is used for verifying the instance transfer learning.
5. Although this paper involves one source dataset, this method can easily be generalized to multiple source datasets.

## Conclusions

Currently, most of the diagnoses of Alzheimer’s disease just made use of the target dataset, and did not consider to make use of the other relevant dataset (subjects, or samples) to improve the classification accuracy. The main contributions of this paper are: (1) A method based on Instance Transfer learning (ITL) is proposed in this paper. The method can transfer the source dataset to target dataset, thereby improving the sample size of the target dataset, different from other relevant transfer learning algorithms. (2) For the relevant datasets with different features, a feature growing algorithm is proposed and can effectively expand the samples of the target domain. (3) The experimental results show that the classification accuracy can be improved apparently. In some cases, this improvement even exceeds 10%. The idea and method of this paper can provide a solution for other studies about small sample problems. Besides, the proposed method is not restricted to specific classifier, or feature learning method, so they are heuristic to relevant researchers.

## Appendix

**Table:**

2 shape features	Hippocampus (L&R)
30 texture features	Contrast(L&R),Correlation(L&R),Energy(L&R),Entropy(L&R),InverseDifferenceMoment(L&R), ShortRunEmphasis(L&R),LongRunEmphasis(L&R),GreyLevelNonuniformity(L&R), RunLengthNonuniformity(L&R),LowGreyLevelRunEmphasis(L&R),HighGreyLevelRunEmphasis(L&R), ShortRunLowGreyLevelEmphasis(L&R),ShortRunHighGreyLevelEmphasis(L&R),LongRunLowGreyLevelEmphasis(L&R), LongRunHighGreyLevelEmphasis(L&R)

## Abbreviations

AD: Alzheimer’s disease
NC: normal control
MCI: mild cognition impairment
MRI: magnetic resonance imaging
SVM: support vector machine
ITL: instance transfer learning
